# Visual category representations in the infant brain

**DOI:** 10.1016/j.cub.2022.11.016

**Published:** 2022-12-19

**Authors:** Siying Xie, Stefanie Hoehl, Merle Moeskops, Ezgi Kayhan, Christian Kliesch, Bert Turtleton, Moritz Köster, Radoslaw M. Cichy

**Affiliations:** 1 Department of Education and Psychology, Freie Universität Berlin, Habelschwerdter Allee, Berlin 14195, Germany; 2 Faculty of Psychology, Department of Developmental and Educational Psychology, University of Vienna, Liebiggasse, Wien 1010, Austria; 3 Max Planck Institute for Human Cognitive and Brain Sciences, Stephanstraße, 04103 Leipzig, Germany; 4 Department of Developmental Psychology, University of Potsdam, Karl-Liebknecht-Straße, 14476 Potsdam, Germany; 5 Institute of Psychology, University of Regensburg, Universitätsstraße, 93053 Regensburg, Germany; 6 Berlin School of Mind and Brain, Humboldt-Universität zu Berlin, Unter den Linden, 10099 Berlin, Germany; 7 Einstein Center for Neurosciences Berlin, Charité-Universitätsmedizin Berlin, Charitéplatz, 10117 Berlin, Germany; 8 Bernstein Center for Computational Neuroscience Berlin, Humboldt-Universität zu Berlin, Unter den Linden, 10099 Berlin, Germany

**Keywords:** infant cognition, cognitive development, visual perception, object recognition, spectral characterization, multivariate analysis, deep learning

## Abstract

Visual categorization is a human core cognitive capacity^1^^,^^2^ that depends on the development of visual category representations in the infant brain.^3^^,^^4^^,^^5^^,^^6^^,^^7^ However, the exact nature of infant visual category representations and their relationship to the corresponding adult form remains unknown.^8^ Our results clarify the nature of visual category representations from electroencephalography (EEG) data in 6- to 8-month-old infants and their developmental trajectory toward adult maturity in the key characteristics of temporal dynamics,^2^^,^^9^ representational format,^10^^,^^11^^,^^12^ and spectral properties.^13^^,^^14^ Temporal dynamics change from slowly emerging, developing representations in infants to quickly emerging, complex representations in adults. Despite those differences, infants and adults already partly share visual category representations. The format of infants’ representations is visual features of low to intermediate complexity, whereas adults’ representations also encode high-complexity features. Theta band activity contributes to visual category representations in infants, and these representations are shifted to the alpha/beta band in adults. Together, we reveal the developmental neural basis of visual categorization in humans, show how information transmission channels change in development, and demonstrate the power of advanced multivariate analysis techniques in infant EEG research for theory building in developmental cognitive science.

## Highlights

• Visual category representations show a developmental path from infants to adults
• In timing from late and slow-evolving to early and fast-evolving
• In format from lower- to higher-complexity visual features
• In spectral signature from the theta to the alpha/beta frequency

## Teaser

Visual categorization depends on the development of visual category representations in the infant brain. Xie et al. reveal the nature and developmental trajectory of visual category representations in 6- to 8-month-old infants toward adult maturity in their key properties of temporal dynamics, format, and spectral signature.

## Results and discussion

The ability to recognize and categorize visual objects effortlessly and within the blink of an eye is a core human cognitive capacity^1^^,^^2^ that develops through learning and interaction with the environment. Behavioral research in infants using looking times^6^^,^^7^^,^^15^ and neural markers of attention provides evidence for visual category processing^16^ and learning^5^^,^^17^ already within the first year of life.

In adults, fundamental research in human and non-human primates has described the nature of the neural representations underlying mature visual categorization abilities, revealing their temporal dynamics,^2^^,^^9^ what features they encode,^10^^,^^11^^,^^12^ their cortical locus,^11^^,^^18^ and how they relate to neural oscillations.^13^^,^^14^ In contrast, these key characteristics of visual category representations^19^^,^^20^^,^^21^^,^^22^^,^^23^ are less well understood in infants due to strong methodological challenges in human and non-human infant neuroimaging research.^8^^,^^24^^,^^25^ In particular, research using EEG—the workhorse of infant neuroimaging for decades—has yielded insights that are principally limited in two ways. One research approach focused on assessing the successful outcome of visual categorization rather than the underlying representations themselves.^26^ Thus, the insights gained about representations are indirect. Another research approach did assess underlying representations directly but was limited to the category of faces^3^^,^^27^ for which known neural markers exist. Thus, the generalizability from the unique and small stimulus subset to the broad set of visual categories of the visual world remains unclear.

Here, we overcome this double impasse to reveal the nature of general visual category representations for various object categories in 6- to 8-month-old infants using EEG data. We do so by leveraging an integrated multivariate analysis framework of multivariate classification^9^ and direct quantitative comparison^28^ of the infant to the adult EEG data and deep learning models of vision.^12^

### The temporal dynamics of visual category representations

**Figure 1:**
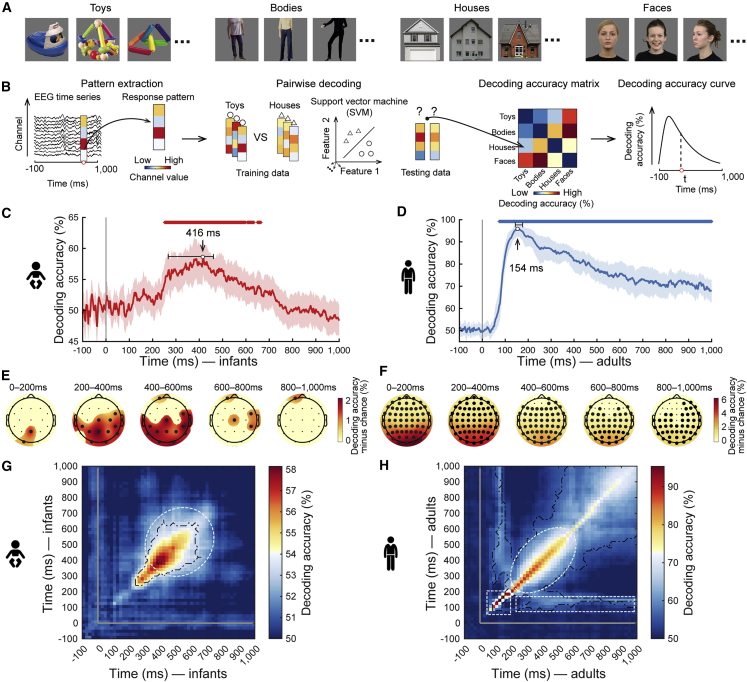
Experimental design and results of time-resolved multivariate analysis (A) The stimulus set comprised 32 cut-out images from four categories each: toys, headless bodies, houses, and faces (full set see OSF Repository at https://osf.io/ruxfg/). (B) Time-resolved multivariate analysis on EEG data. First, we extracted condition-specific EEG sensor activation values for every time point in the epoch and formed them into response vectors. Then, using a leave-one-out cross-validation scheme, we trained and tested a support vector machine to classify visual object categories from the response vectors. The results (pairwise decoding accuracy, 50% chance level) were aggregated in a decoding accuracy matrix of size 4 × 4, indexed in rows and columns by the conditions classified. The matrix is symmetric along the diagonal, and the diagonal is undefined. Averaging the lower triangular part of the matrix resulted in grand average decoding accuracy as a measure of how well visual representations discriminate categories at a particular time point. (C and D) The grand average time course of visual category decoding in infants (C) and adults (D). The gray vertical line indicates onset of image presentation. Shaded margins indicate 95% confidence intervals (CIs) of decoding accuracy. Horizontal error bars indicate 95% CIs of peak latency. Rows of asterisks indicate time points with significantly above-chance decoding accuracy (infant n = 40 or adult n = 20, right-tailed sign permutation tests, cluster-defining threshold p < .005, corrected significance level p < .05). Detailed statistical information is listed in Table S1A. For visualization of single participant infant data see https://github.com/siyingxie/VCR_infant. (E and F) Results of category classification in EEG channel-space searchlight analysis for infants (E) and adults (F). Bold dots indicate the EEG channels with significantly above-chance decoding accuracy (right-tailed sign-permutation tests, p < .05, FDR-corrected). (G and H) Results of time-generalization analysis for infants (G) and adults (H). Detailed statistical information is listed in Table S1E. For visualization of single participant infant data see https://github.com/siyingxie/VCR_infant. The gray vertical and horizontal lines indicate the onsets of image presentation. Black outlines indicate time point combinations with significantly above-chance decoding accuracy (right-tailed sign permutation tests, cluster-defining threshold p < .005, corrected significance level p < .05). See also Figure S1 and Table S1.

Infant participants (n = 40) viewed 128 images of real-world objects from four categories (i.e., toys, bodies, houses, and faces, see Figure 1A; for rationale of category choice see Method details) while we acquired EEG data. The age group was chosen based on extensive work showing that by this age infants reliably discriminate between basic level categories .^26^^,^^29^^,^^30^^,^^31^ Images were presented for 2 s every 2.7–2.9 s. For direct comparison we acquired EEG data in adult participants (n = 20) viewing the same stimulus set with an adapted experimental design (Figures S1A and S1B). We consider the epoch of −100 ms to +1,000 ms with respect to stimulus onset in our analyses.

To reveal the time course with which visual category is discriminated by visual representations, we used time-resolved multivariate pattern analysis^9^ (Figure 1B). We report peak latency (95% confidence intervals [CIs] in brackets) as the time point during neural processing when category information was most explicit, as well as onset and offset of significance for each group.

In infants (Figure 1C), the classification curve rose gradually from 100 ms onwards, reaching significance at 252 ms (250–254 ms), followed by a broad peak at 416 ms (268–462 ms) and a gradual decline. This pattern of result did not depend on any particular object or category (Figures S1E and S1F), held equally for classifications within and across the animacy division (Figures S1G and S1H), and emerged equivalently for alternative common analysis schemes (Figures S1I–S1K). In contrast, in adults, the classification curve had a different shape (Figure 1D). It emerged earlier (significant at 72 ms [72–74 ms]) and faster, peaking at 154 ms (144–176 ms), compared to infants (p < .001, bootstrap test, Table S1A). The observed delay is not only due to longer latencies already at the early cortical processing stages: the P100 component peak in infants was delayed by 22–68 ms (Figures S1C and S1D, Table S1F), consistent with previous studies.^6^^,^^32^^,^^33^^,^^34^ Instead, the grand average ERP peak was much stronger, delayed by 98–242 ms. This suggests that the observed peak latency differences with which category representations emerge reflect a mixture of processing delays at early and late processing stages.

Searchlight analysis in EEG channel space revealed that information about visual category representations was highest in EEG channels overlying occipitoparietal cortex in both infants (Figure 1E) and adults (Figure 1F), tentatively suggesting partly similar cortical sources in the posterior cortex.

This multivariate approach constitutes a novel analytical access point to visual category representations in infants from EEG data. Noteworthy, there is no simple mapping function of our results to the results of classical univariate results, as the approaches differ in many aspects. Univariate analyses focus on single electrodes or averages, while multivariate analyses focus on patterns across electrodes, potentially increasing sensitivity.^35^^,^^36^ In adults, univariate and multivariate analyses also do not directly agree.^9^^,^^37^ However, the multivariate results carry meaning, as they can be meaningfully related to behavior.^9^^,^^38^ Further research combining multivariate analyses with behavioral measures^7^^,^^15^ in infants is needed.

The rise and fall of the classification curves in a few hundred milliseconds might indicate rapid changes in the underlying visual representations,^2^^,^^9^ slow ramping up of persistent representations, or a combination of both. To investigate this, we assessed the temporal stability of visual representations using time-generalization analysis.^39^ We determined how well classifiers trained on predicting visual category from EEG at one time point perform when tested at other time points. Lack of generalization across time indicates transience of the underlying visual representation, whereas generalization across time indicates persistence.

In both infants (Figure 1G) and adults (Figure 1H), classification accuracy was highest along the diagonal (i.e., similar time points for training and testing) with a broadening over time (white dotted ellipse). This result suggests common neural mechanisms of a rapid sequence of processing steps that result in an outcome held online for further use, indicated by rapidly changing transient representations at earlier time points and more slowly changing persistent representations at later time points, respectively.

In addition to this general similarity between infants and adults, two notable differences were indicative of incomplete development of feedforward and feedback information processing in infants. For one, early after stimulus onset, when neural processing is dominantly feedforward, in adults we observed high classification accuracy trailing the diagonal narrowly (Figures 1H, 50–200 ms, dotted square), indicating rapidly changing representations. Infants did not exhibit such signals. This pattern suggests incomplete development of feedforward visual information processing mechanisms in infants. Second, in adults, the classifier generalized well for the time point combination of 100–200 ms and 200–1,000 ms (Figure 1H, white striped rectangle). This suggests highly persistent representations, likely emerging in the early visual cortex.^9^ There were no such signals in infants. This indicates incomplete neural structures for recurrent processing that maintain visual information online for long stretches of time.

While the overall pattern of results did not depend on any particular category (Figures S1L and S1M), we cannot exclude that differences between age groups could also be due to differences in experimental task or signal-to-noise ratio (SNR). By design, we boosted SNR in adults compared to infants to increase the chance of identifying similarities at the cost of interpretative difficulties for differences. These difficulties are, however, alleviated by focusing on peaks as core measures for interpretation whose size, but not latency, depends on SNR.

### Shared visual category representations between infants and adults

**Figure 2:**
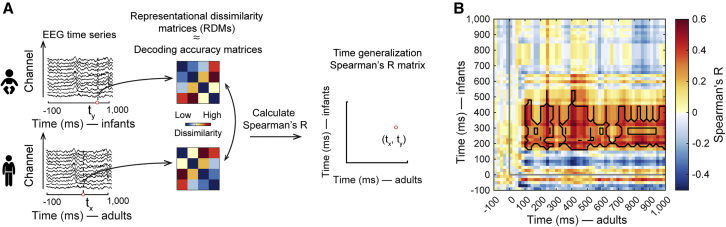
Category representations shared between infants and adults (A) We used RSA to relate category representations in infants and adults. We interpret decoding accuracy as a dissimilarity measure on the assumption that the more dissimilar two representations are, the better the classifier performs. This allowed us to use time-resolved decoding accuracy matrices as representational dissimilarity matrices (RDMs) that summarize representational similarities between category representations. We compared RDMs (Spearman’s *R*) in infants (average across participants) and adults (for each participant separately) for all time point combinations (t_x_, t_y_), assigning the values to a time-generalization matrix indexed in rows and columns by the time in adults (t_x_) and infants (t_y_). (B) Average time-generalization matrix relating category representations in infants and adults over time. Detailed statistical information is listed in Table S2A. For visualization of single participant data see https://github.com/siyingxie/VCR_infant. The gray lines indicate image onset. Black outlines indicate time point combinations with significant correlation (n = 20, right-tailed sign permutation tests, cluster-defining threshold p < .005, corrected significance level p < .05). See also Figure S2 and Table S2.

The results so far show that we identified visual category representations in both infants and adults and that their time courses have both similar and different aspects. However, we have not tested whether infants and adults have similar category representations. An alternative hypothesis is that we observe time courses of category classification for infants and adults, but those are unrelated rather than shared representations. Direct identification of shared representations between infants and adults is challenging due to differences in the time course over which the representations emerge and the EEG channel spaces differ. We used a time-generalization variant of representational similarity analysis (RSA)^28^ (Figure 2A) to overcome these hurdles. In short, we abstracted multivariate signals from the incommensurate infant and adult EEG channel spaces to a common representational dissimilarity space, and we compared the signals across all time point combinations.

We observed similarity in visual category representations between infants and adults at the time point combinations of 160–540 ms in infants, and 100–1,000 ms in adults (Figure 2B, peak latency in infants: 200 ms [200–360 ms]; in adults: 120 ms [120–1,000 ms]). This result was similarly achieved for alternative processing and data aggregation choices (Figures S2A and S2B), and did not depend on any particular category except on toys (Figure S2C). Our findings establish quantitatively and directly that infants and adults share visual category representations.

In sum, the emerging picture is one of not yet fully developed dynamics of adult-like visual category representations in infants. Representations in infants emerged later, slower, and lacked particular components of feedforward and recurrent processing, possibly related to immature myelination^40^ and synaptic connectivity.^41^ Nevertheless, representations in infants and adults shared large-scale temporal dynamics that encoded visual category information similarly, consistent with previous studies showing partly adult-like behavioral^3^^,^^5^^,^^7^^,^^15^ and neural^19^^,^^20^ category sensitivity in the first year of age.

Our approach goes beyond previous EEG work in developmental visual neuroscience in three ways. First, rather than relying on indirect inference from attentional markers indicating successful categorization,^26^ our approach assessed representations directly as they emerge with millisecond resolution. Second, our approach is not limited to the face category and face-specific EEG components,^3^^,^^27^ but allows the study of potentially any visual category. Third, our approach enabled a new quantitative comparison^28^ of infant and adult visual category representations.

Our results make direct predictions for the detailed developmental trajectory of visual category representations.^42^ We expect category representations to emerge increasingly earlier and with faster temporal dynamics with increasing age, with additional feedforward and feedback components appearing at critical stages until a mature adult-like system emerges. Our approach makes these predictions immediately testable in future studies using other age groups between early infancy and adulthood.

More broadly, our multivariate EEG analysis approach demonstrates a novel access point to largely unmapped neural representations in the infant brain, with strong potential to inform theories of cognitive development for cognitive capacities that emerge in the first year of life, such as object learning,^5^^,^^6^^,^^17^ speech processing,^43^ and core knowledge systems.^44^ Combined with human infant fMRI^19^^,^^20^^,^^25^ and behavioral assessment^15^ in a common framework,^45^ this promises to reveal the unknown spatiotemporal neural dynamics underlying cognitive functions in infants in the future.

### The format of visual category representations

The time-resolved multivariate pattern analysis revealed the presence and dynamics of visual category representations in the infant and adult brain. However, by itself, it is unable to specify their format, i.e., what type of visual features they encode. We hypothesized that adults would encode visual features represented at all levels of the visual processing hierarchy from low- to high-complexity.^46^ Instead, infants would encode visual features rather of low- and mid-complexity, as predicted from visual behavior^7^^,^^26^ and anatomical development patterns^42^ of the infant visual brain.

**Figure 3:**
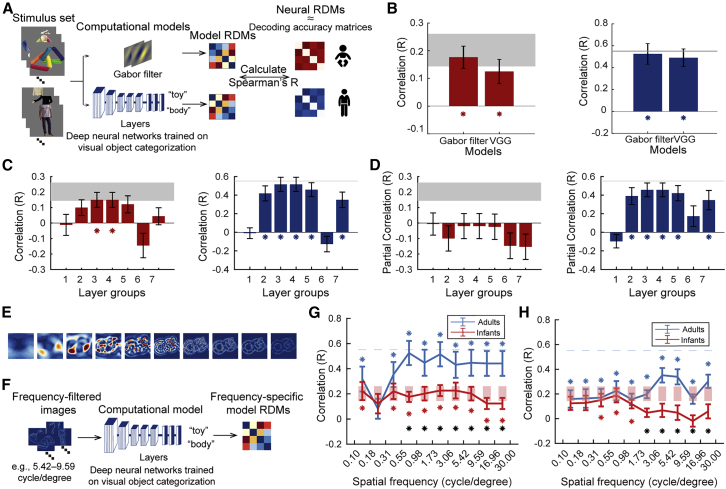
The format of category representations in infants and adults (A) We characterized what type of visual features are encoded in category representations in infants and adults by relating them to computational models using RSA. We ran the stimulus images through a Gabor filter model and the VGG-19 deep neural network trained on object categorization. We constructed RDMs from their unit activation patterns (visualized in https://github.com/siyingxie/VCR_infant). We then compared model RDMs to infant and adult neural RDMs (constructed as the average of RDMs over time from 95% CIs around peak latency of time-resolved category classification, see Figures 1C and 1D). (B) Results for infants (left) and adults (right) at the whole-model level. Error bars represent standard errors of the mean. Asterisks indicate significant correlation (infant n = 40, adult n = 20, two-tailed sign-permutation tests, p < .05, FDR-corrected). (C) Results for infants (left) and adults (right) at the deep convolutional neural network (DNN) layer level. Error bars represent standard errors of the mean. Asterisks indicate significant correlation (infant n = 40, adult n = 20, two-tailed sign-permutation tests, p < .05, FDR-corrected). For (B) and (C), statistical details (i.e., correlations and p values) are in Table S3A. (D) Results for infants (left) and adults (right) at the DNN layer level after removing the effect of the other age group respectively by partialling out the average RDM. Error bars represent standard errors of the mean. Asterisks indicate significant correlation. (E) Example of Butterworth-filtered images in different spatial frequencies. (F) We characterized visual features encoded in visual representations in terms of spatial frequency content. We ran the frequency-filtered images through the VGG-19 DNN. We constructed spatial-frequency-specific RDMs from the DNN unit activation patterns. We then compared model RDMs to infant and adult neural RDMs as described in (A). (G) Relating frequency-specific image content to neural representations. The results indicate a significant correlation across all frequencies (except one bin at 0.18 cycle per degree [cpd]) in both infants and adults, with higher correlations for adults than infants above 1 cpd. (H) Spatial-frequency-specific results for infants (blue curve) and adults (red curve) at the whole-model level. For (G) and (H), asterisks are color-coded as result curves indicate statistical significance (infant n = 40, adult n = 20, two-tailed sign-permutation tests, p < .05, FDR-corrected); black asterisks indicate significant difference between age groups (two-tailed Mann-Whitney U tests, p < .05, FDR-corrected). See also Figure S3 and Table S3.

To determine the format of category representations, we related them to computational models of vision (Figure 3A). We probed two types of models: a Gabor filter model as a model of simple visual features,^47^ and the deep neural network VGG-19 model^48^ trained on object categorization, which exhibits a hierarchy of low-to-high complexity features along with its layers, and predicts activity along the visual processing hierarchy of the adult human brain well.^12^^,^^49^

Assessing first the Gabor model and an aggregated summary of the VGG model across layers, we found similar representations between both models and infant and adult visual representations (Figure 3B). This suggested that features ranging from low to high complexity might contribute and invited further in-depth analysis.

Turning to the VGG model first, we conducted a finer investigation of VGG at the level of layers. Considering each layer separately, we found that in infants, middle layers predicted brain activity best, with layer groups 3 and 4 being significant (Figure 3C, left). In contrast, layers at all stages were significantly predictive in adults (Figure 3C, right). This pattern of results was also achieved for other types of deep neural network architectures (Figures S3A and S3B) and independent of data selection choices (Figures S3C and S3D), demonstrating the robustness of the result. This pattern of results suggests that infants and adults share similar visual features with the VGG model at intermediate complexity. We ascertained this in two ways. First, using partial correlation, we related the VGG model to each age group while partialling out the effect of the other age group. This abolished all effects in infants (Figure 3D, left) while leaving the resulting pattern in adults unchanged (Figure 3D, right), suggesting that the features underlying visual category representation in infants are a subset of the features in adults. Second, we conducted a variance partitioning analysis between the VGG model and infant and adult visual representations at layers 3 and 4, revealing shared variance (both *R*^2^ = 0.16; p < .05, FDR-corrected). This reveals that in infants, category representations are in the format of low- to intermediate-complexity features and form a subset of the representations seen in adults, whereas in adults category is discriminated by features at all levels of complexity.

The prediction by the Gabor filter model as well as early layers of VGG in adults suggests that in both age groups category is discriminated by representations encoding features of intermediate and low complexity, albeit to a different degree or in different ways. This is consistent with observations that low-level visual features are represented in high-level ventral visual cortex alongside features of higher complexity,^50^^,^^51^^,^^52^ and that categories are systematically related to category through differences in spatial frequency content, thus supporting classification.^53^

We thus investigated the role of low-level features at different spatial frequencies in visual category representations in infants and adults. We filtered the stimulus material in spatial frequency in 100 bins spaced logarithmically between 0.1 and 30 cycles per degree (cpd) visual angle (for an example see Figure 3E). As expected, visual object categories were associated with different spatial frequency content in the images (Figure S3E) that allows category to be determined directly from the images (Figure S3F). Using RSA, we assessed the similarity between category representations and the spatially filtered images. We observed a significant relationship across all spatial frequencies (except at 0.18 cpd) in both infants and adults (Figure 3G), with stronger relationships for adults than infants above 1 cpd. This shows that category representations in infants and adults are differentiated by features across the spatial frequency spectrum, with a stronger role of higher spatial frequencies in adults.

Based on this result, we refined the deep neural network model-based analysis with respect to spatial frequency. We compared the VGG model’s representation of the filtered images with infant and adult category representations (Figure 3H). For adults, the result revealed similar representations across all spatial frequencies as expected, with a peak at 3.06–5.42 cpd. In contrast, for infants, the similarity was restricted to spatial frequencies from 0.31 to 1.73 cpd, with a peak at 0.55–0.98 cpd. This is consistent with the shift in peaks in spatial sensitivity from low spatial frequency up to 1 cpd^54^^,^^55^ to higher spatial frequency at 2–6 cpd.^56^ As expected from the previous analysis, we find significantly stronger correlations for higher spatial frequencies in adults than in infants.

Taken together, this reveals that in infants, category representations are in the format of low- to intermediate-complexity features at low spatial frequency and form a subset of the representations seen in adults. In contrast, in adults, category is discriminated by features at all levels of complexity and all spatial frequencies, with a higher reliance on high spatial frequencies.

Which processes may contribute to the emergence of high-complexity features in the developmental trajectory from infancy to adulthood? At this moment, we can only speculate. The results of the time-generalization analysis in conjunction suggest that local and far-reaching feedback processes from the frontal cortex might be involved.^57^^,^^58^^,^^59^ In more cognitive terms, linguistic and semantic processing is known to modulate visual processing in adults and might modulate visual representations.^60^

Previous research investigating the format of infant visual category representations tested hypotheses one by one through experimental manipulation; for example, determining whether infants are sensitive to stimulus inversion^27^ or tolerant to changes in viewing conditions.^61^ Instead, our approach allows the comparison of any number of hypotheses as captured in explicit, image-computable computational models to predict infant visual category representations increasingly well. To speed up this process, we make the data publicly available.

Our findings further suggest constraints for artificial intelligence research. The biological brain inspired the engineering of deep learning models, but the models’ learning has remained biologically unrealistic^12^^,^^49^^,^^62^ and is perceived as a major impediment to building better models. We suggest that models of human visual categorization striving for increased biological realism should follow a similar developmental trajectory of representations as described here.

### Spectral properties of visual category representations

**Figure 4:**
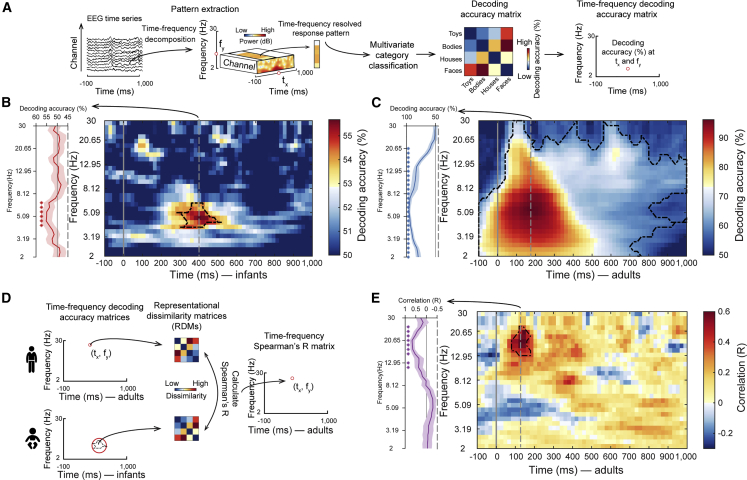
Spectral characterization of infant and adult category representations (A) Category classification based on frequency-resolved EEG data. We first decomposed EEG data in time and frequency using *Morlet* wavelets for each trial and each channel, yielding a trial-wise representation of induced oscillatory power. We then conducted the time-resolved multivariate classification of category separately on each frequency bin. This yielded a 4 × 4 matrix of decoding accuracies at each time point and frequency bin, which we either averaged to obtain grand average category classification, (B) and (C), or used as an RDM in RSA, (D) and (E). (B and C) Results of time- and frequency-resolved multivariate analysis for infants (B) and adults (C). Detailed statistical information is listed in Table S4A. (D) RSA procedure linking oscillation-based visual category representations in infants and adults. We first created a single aggregate infant oscillatory RDM by averaging decoding accuracy matrices based on the extent of the cluster in the infant data. We compared (Spearman’s *R*) this aggregate infant RDM to time- and frequency-resolved RDMs for each participant in the adult sample. This yielded a two-dimensional matrix indicating in which frequency range and when category representations are similar between infants and adults. (E) Similarity between infant theta-based category representations and adult category representations resolved in time and frequency. Detailed statistical information is listed in Table S4B. For (B), (C), and (E), for the single participant data see https://github.com/siyingxie/VCR_infant; the gray vertical lines indicate the onset of image presentation; line profiles of classification accuracy or correlation at the peak latency (gray vertical dashed lines) were shown on the left of the plots, respectively; black outlines indicate time point combinations with significant results (infants n = 40, adults n = 20, right-tailed permutation test, cluster-defining threshold p < .005, corrected significance level p < .05). See also Figure S4 and Table S4.

Neural oscillations underlie the formation and communication of visual representations.^14^^,^^63^^,^^64^^,^^65^ Here, we determined the spectral signature of visual category representations in infants as a first step toward describing their relationship to neural oscillations. For this, we resolved EEG data in distinct frequency bins from 2 to 30 Hz and performed time-resolved visual category classification on each bin separately (Figure 4A). In infants (Figure 4B), we observed significant category classification accuracy in a specific cluster in the theta band with a peak at 4.63 Hz (2.91–6.73 Hz) and 400 ms (160–580 ms). This result reveals activity in the theta band as the spectral signature of visual category representations in infants. In contrast, in adults (Figure 4C), the cluster extended across the whole frequency range and time course investigated. It shows that the spectral signature of visual category representations in adults is broadband. The patterns of results did not depend on any particular category except for faces in infants (Figures S4E and S4F). Note that the observed differences in infants and adults are not a trivial consequence of differences in EEG power spectra pattern or category, as those were similarly broadband in both infants and adults and for all categories (Figures S4A and S4B). Further, the classification peaks do not map onto the power spectra peaks in terms of frequency and latency (Figures S4C and S4D). They are thus not a function of SNR in the power spectrum. Finally, the difference between infants and adults is not due to higher inter-subject variability in spectral power patterns in adults, as different measures of variability were lower in adults than in infants (Table S4D).

The observed pattern of results is consistent with two alternative hypotheses about the relationship between the oscillatory basis of visual category representations in infants and adults. One hypothesis is that there is a direct match in frequency, suggesting that peak classification in infants and adults is at similar frequencies (i.e., at 4.63 Hz and 5.59 Hz, respectively). Another hypothesis is an upward shift across age, made plausible by the observations that brain rhythms increase in frequency during infant development.^66^

To arbitrate between those hypotheses, we determined which frequency band and time points category representations in adults were similar to infant category representations identified in the theta band (Figure 4D). We extracted representational dissimilarity matrices (RDMs) from the infant data at the cluster in the theta range. We used their average as a search template, comparing it to RDMs from the adult data for all time point and frequency combinations. We found a cluster of significant correlations with a peak at 17.13 Hz (9.78–20.65 Hz) at 120 ms (120–360 ms) (Figure 4E). This pattern of results was partly independent of category (except faces, Figure S4G), held across different data aggregation schemes and ways to assess the theta cluster (Figure S4H). Further, there was no relationship between signals at the (non-significant) peak in infant alpha/beta at 100 ms and adult signals at any time-frequency combination (Figures S4I and S4J). It directly demonstrates, both specifically and quantitatively, an upward shift in the spectral signature of neural activity supporting visual category representations from the theta range in infants to the alpha/beta range in adults. As expected from the investigation of the representational format above, the shared representations which shifted relied on low spatial frequency features (Figures S3G and S3H).

The shift observed is not a trivial consequence of differences in the peak latency and frequency of the power spectrum, which are similar in infants and adults (Figures S4A–S4D). Closer inspection of the classification results (Figures 4B and 4C) at peak latency reveals a similar peak around 4.63–5.59 Hz (Figures 4B and 4C, line profiles), leaving open the possibility that to some degree the profile observed in infants might be a down-scaled and noisier version of the situation in adults, and predicting shared representations across age group in the theta band. Instead, the shared representations are present only in the alpha/beta band (Figure 4E, line profile), demonstrating a clear dissociation from overall signal strength.

One interpretation of these findings is that in infants, neural networks for learning and memory associated with the theta rhythm contribute to the formation of category representations,^67^^,^^68^ whereas in adults, equivalent category representations are processed quickly in fully developed semantic networks associated with the alpha/beta rhythms.^69^^,^^70^ Alternatively, the frequency shift might be due to more efficient axonal transmission as a result of improved myelination^40^ that may enable higher neural oscillations to emerge in the same neural circuits, consistent with increases in the prevalent frequency in the EEG across development.^66^ On this account, our finding suggests a novel general developmental trajectory for neural communication channels in the human brain: specific information-processing mechanisms working at low frequency in infants are shifted up in frequency gradually across development, and the size of this shift depends on the differences in neural circuit myelination. However, we note that power in a frequency does by itself indicate oscillations in that frequency, and further research is needed to establish this firmly, e.g., by distinguishing aperiodic from periodic components.^71^^,^^72^ Similarly, we do not observe a one-to-one mapping between shared representations revealed by time-resolved analysis (Figure 2B) and time-frequency resolved analysis (Figure 4E). Instead, we expect the relationship to be akin to the complex relationship between neural oscillations and evoked responses.^73^^,^^74^ Further research is needed to resolve to which degree they depend on distinct or shared neural phenomena.

### The nature and developmental trajectory of infant to adult visual category representations

In sum, our results reveal the nature and developmental trajectory of the infant to adult visual category representations, from infancy to adulthood. Temporal dynamics change from slowly to quickly emerging in time, the format from visual features of low and intermediate complexity to features of high complexity, and the oscillatory signature from the theta to the alpha/beta frequency. These results provide insight into visual category representations that underlie the development of fast and efficient visual categorization skills in humans. They also further reveal how cortical information transmission channels change in human development and demonstrate the power of advanced multivariate analysis techniques in infant EEG research for developmental cognitive science.

## STAR★Methods

### Key resources table

**Table:** 

REAGENT or RESOURCE	SOURCE	IDENTIFIER
**Deposited data**		

Raw and analyzed data	This paper	https://osf.io/ruxfg/

**Software and algorithms**		

Customized code	This paper	https://github.com/siyingxie/VCR_infant
MATLAB	Mathworks Inc.: https://www.mathworks.com/	https://www.mathworks.com/products/matlab.html; RRID: SCR_001622
Psychtoolbox-3	Psychtoolbox: https://www.psychtoolbox.net/	http://psychtoolbox.org/; RRID: SCR_002881
Fieldtrip Toolbox	FieldTrip: https://www.fieldtriptoolbox.org/	https://www.fieldtriptoolbox.org/; RRID: SCR_004849
Brainstorm3	Brainstorm: https://neuroimage.usc.edu/brainstorm/	https://neuroimage.usc.edu/brainstorm/; RRID: SCR_001761
LIBSVM: A library for Support Vector Machines	LIBSVM: https://www.csie.ntu.edu.tw/∼cjlin/libsvm/	https://www.csie.ntu.edu.tw/∼cjlin/libsvm/; RRID: SCR_010243
MatConvNet: CNNs for MATLAB	MatConvNet: https://www.vlfeat.org/matconvnet/	https://github.com/vlfeat/matconvnet

### Resource availability

#### Lead contact

Further information and requests for the resources should be directed to and will be fulfilled by the lead contact, Radoslaw M. Cichy (rmcichy@zedat.fu-berlin.de).

#### Materials availability

This study did not generate new unique reagents.

### Experimental model and subject details

Two independent pools of participants took part in this study: 6–8 months old infants and young adults. We chose this infant age group based on extensive evidence from behavioral and electrophysiological work showing that infants discriminate between various basic level visual categories by this age, whereas in younger infants, category discrimination is less stable and relies more on the chosen paradigm and the specific categories assessed.^26^^,^^29^^,^^30^^,^^31^ The infant sample was assessed at the Max Planck Institute for Human Cognitive and Brain Sciences in Leipzig, Germany. It comprised 48 participants, of which 8 were excluded due to insufficient data, yielding a final sample analyzed of 40 infant participants (gender: 19 female, age: mean ± SD: 214.9 ± 14.76 days). The adult sample was assessed at the Freie Universität Berlin, Germany. It comprised 20 participants of which none was excluded (gender: 11 female, age: mean ± SD: 26.1 ± 3.81 years). Caregivers of all infants and all adult participants gave written informed consent. The study was conducted according to the Declaration of Helsinki and the infant and adult protocols were approved by the respective local ethic committees.

### Method details

#### Stimuli

The stimulus set consisted of 32 object images in each of the four categories included: houses, toys, faces, and bodies. We chose those categories for four reasons: (1) they are highly familiar to infants, and infants encounter them in everyday life; (2) faces,^75^^,^^76^^,^^77^^,^^78^ toys,^3^ bodies,^79^^,^^80^ and houses^81^^,^^82^ (as large objects that define scenes) have been used in previous infant research making our results in principle comparable; (3) they have well-described and distinct neural signatures in adults^83^; and (4) a recent fMRI study showed distinct neural signatures for faces, objects, and scenes in infants, too.^20^ This yielded a total set of 4 × 32 = 128 object images. All object images were cut-out from color photographs. All images are available in the OSF repository at https://osf.io/ruxfg/. We analyzed the data at the level of category.

#### Experimental procedure in the infant sample

In the infant experiment, participants were presented with 272 trials divided into four blocks. Each block had the same basic structure. At the beginning of each block four stimuli (one stimulus per object category) were separately presented three times in randomized order. Thereafter participants were presented with a random sequence of images comprising the same four stimuli seven more times, intermixed with 28 other images (seven images per category) presented only once. This experimental design was chosen because it allows assessing the effect of object image repetition of infant brain responses, but this question is orthogonal to the ones pursued here and will be reported separately.

Each trial consisted of a fixation dot presented for a variable duration of 700–900 ms, followed by a stimulus presented for 2,000 ms at the center of the screen (Figure S1A). To capture the attention of the infants and direct their gaze to the screen we implemented two measures. First, we presented a yellow duck image and duck sound for 1,000 ms at the beginning of each block and thereafter every 10 trials. Second, each stimulus was presented together with one of ten arbitrary sounds that were assigned randomly at each trial.

During the assessment, infants sat on their care giver’s lap at a viewing distance of about 80 cm from a 17-inch. CRT screen. The object images were presented at the center of the screen, subtending a visual angle of approximately 5.0°. To monitor infants’ gaze, we recorded videos of infants’ faces throughout the experiment.

#### Experimental procedure in the adult sample

We adapted the experimental design for the adult sample. In short, all images were shown equally often, with higher number of repetitions, at shorter presentation times and higher presentation rates that in the infant study (Figure S1B).

The first 3 participants were presented with 1,280 trials divided into 5 runs. In each run each object image was presented twice. The other 17 participants were presented with 3,840 trials divided into 10 runs. In each run each object image was presented three times. In each run, images were presented in random order, and runs were separated by breaks that were self-paced by the participants.

Each trial consisted of the presentation of a fixation cross with a variable duration of 600–800 ms, followed by a stimulus presentation for 500 ms. Stimuli were presented at the center of the screen at a visual angle of approximately 7.0°.

Participants were instructed to keep fixation on the center of the screen throughout the experiment. To ensure that participants attended to the stimuli and to avoid contamination of the relevant recording times with blink artefacts, participants were instructed to press a button and blink their eyes in response to a paper clip image that was shown randomly every 4 to 6 trials (average 5 trials). Paper clip trials were excluded from all further analysis.

#### EEG acquisition and preprocessing

##### Infant sample

EEG data for the infant sample were recorded in a shielded room using 30 Ag/AgCl ring electrodes and a TMSi 32-channel REFA amplifier at a sampling rate of 500 Hz. Electrodes were placed according to the standard 10-20 system. Electrodes V+Fp2 and V- recorded the vertical electrooculogram (VEOG), and electrodes H-F9 and HF+10 recorded the horizontal electrooculogram (HEOG), Cz served as the online reference. We conducted preprocessing using the Fieldtrip toolbox.^84^ The continuous EEG data was segmented for each trial into epochs. For subsequent time-resolved multivariate analysis we extracted the epoch from −200 ms to +1,000 ms with respect to image onset. For analysis that was additionally resolved in frequency we used longer epochs to allow better estimation at lower frequencies from −500 ms to +1,000 ms.

We removed all trials during which participants did not gaze at the screen for 1,000 ms after stimulus onset as assessed by visual inspection of the video recordings. In this reduced trial set (mean ± SD: 139.6 ± 47.76 trials) we removed noisy channels (mean ± SD: 1.25 ± 1.32) and replaced them by interpolated data from adjacent electrodes. We further conducted independent component analysis (ICA) and removed components related to eye-movement and muscle artifacts as identified by visual inspection.

##### Adult sample

EEG data for the adult sample were recorded using an EASYCAP 64-channel system and a Brainvision actiCHamp amplifier at a sampling rate of 1,000 Hz. Data were filtered online between 0.03 and 100 Hz. Electrodes were placed according to the standard 10-10 system. Electrode Fz served as the online reference. We conducted preprocessing using the Brainstorm 3 toolbox.^85^ Up to two noisy channels were removed for each participant as identified by visual inspection. We conducted ICA to identify and remove eye-movement and muscle artifact components by visual inspection of independent components. The continuous EEG data were then segmented for each trial into epochs from −200 ms to +1,000 ms (for time-resolved analysis) and from −500 ms to +1,000 ms (for time- and frequency-resolved analysis).

#### EEG time-frequency decomposition

We decomposed the EEG time series into frequency-specific components by convolving the data with complex *Morlet* wavelets separately for each trial and sensor. We performed decomposition based on single trials so that the decomposed activity reflects stimulus-locked evoked responses and induced responses.^73^ The wavelets had a constant length of 2,600 ms and were logarithmically spaced in 30 frequency bins between 2 Hz and 30 Hz. We obtained the absolute power values for each time point and frequency bin by taking the square root of the resulting time-frequency coefficients. We normalized these power values to reflect relative changes (expressed in dB) with respect to the pre-stimulus baseline (–300 ms to −100 ms with respect to stimulus onset). We downsampled the time-frequency representations to a temporal resolution of 50 Hz (by averaging data in 20ms-bins) to increase the signal-to-noise ratio of subsequent analyses. This yielded for each trial a power value for each time point and frequency bin.

#### Multivariate classification of visual category from EEG data

To characterize the temporal dynamics with which visual category representations emerge in infant and adult brains we conducted multivariate EEG classification using linear support vector machines (SVMs). We analyzed the infant and the adult data set separately and equivalently.

We conducted two common variants of multivariate EEG classification: time-resolved EEG analysis^9^^,^^37^ and time-generalization analysis.^39^ We conducted the analysis separately on the adult and infant sample, and separately for each participant. All analyses employed binary c-support vector classification (C-SVC) with a linear kernel as implemented in the LIBSVM toolbox.^86^ The details of the time-resolved and the time-generalization analysis are as follows.

#### Time-resolved classification

We used time-resolved multivariate pattern analysis on EEG data (Figure 1B) to determine the time course with which visual category representations emerge in infant and adult brains. For each time point of the EEG epoch (from −200 ms to +1,000 ms), we extracted trial-specific EEG channel activations (i.e., 25 in infants and 63 in adults) and arranged them into pattern vectors for each of the four category conditions (i.e., face, house, body, and toy) of the stimulus set. To increase the signal-to-noise ratio (SNR), we randomly assigned raw trials into four bins of approximately equal size each and averaged them into four pseudo-trials. We used a leave-one-pseudo-trial-out cross validated classification approach. We trained the SVM classifier to pairwise decode any two conditions using three of the four pseudo-trials for training. We used the fourth left-out pseudo-trial for testing, yielding classification accuracy (chance level 50%) as a result. The procedure was repeated 100 times, each time with a new random assignment of trials to pseudo-trials. The resulting decoding accuracy was averaged across repetitions and assigned to a decoding accuracy matrix of size 4 × 4, with rows and columns indexed by the conditions classified. The matrix is symmetric across the diagonal, with the diagonal undefined. This procedure yielded one decoding matrix for every time point.

#### Time-frequency resolved classification

In addition to classifying visual category from broadband responses (i.e., single trial raw unfiltered waveforms), we classified object categories from oscillatory responses. This analysis followed the same rationale as the classification analysis described above, with the only difference that classification was conducted on power value patterns instead of raw activation value patterns. The analysis was conducted separately for each frequency bin separately. This resulted in a decoding accuracy matrix of size 4 × 4 as defined above for every time point and every frequency bin.

#### Time generalization analysis

We used time-generalization classification analysis^39^ to determine how visual representations emerging at different time points during the dynamics of visual perception relate to each other. For time and memory efficiency, we down-sampled the EEG data to a sampling rate of 50 Hz by averaging the raw EEG data in 20 ms bins. The procedure was equivalent to the time-resolved classification analysis with the only difference that classifiers trained on data from a particular time point were not only tested on left out data from the same time point, but iteratively on data from the same and all other time points. The idea is that successful classifier generalization across time points indicates similarity of visual representations over time. This analysis yielded thus a size 4 × 4 decoding accuracy matrix indexed in rows and columns by the conditions compared for all time point combinations from −200 to +1,000 ms. We averaged the entries of the decoding accuracy matrix at each time point, yielding a temporal generalization matrix indexed in rows and columns by training and testing time.

#### Sensor-space searchlight analysis

We performed a sensor-space searchlight analysis^87^^,^^88^ to localize in EEG channel space which channels contributed to the classification of category. For each EEG channel we defined a neighborhood as a sphere of the 10 (for adults) or 5 (for infants) closest EEG channels. For each EEG channel we then performed time-resolved category classification analysis, limiting data entering the analysis to its neighboring channels. Averaging across all pairwise category classifications yielded one decoding accuracy for each time point and for each EEG channel. We further averaged the results in 200 ms bins, yielding a single EEG channel searchlight map of grand average decoding accuracy for each time bin.

#### Computing spatial-frequency specific versions of the stimulus set

To assess the role of spatial frequency on visual object categorizations, we decomposed the stimulus set in terms of spatial frequency. For this, we first used the Fourier transform to transform each image into the frequency domain. We then defined a set of 100 Butterworth band-pass filters (complex higher order filters with a roll-off response rate of 5) logarithmically spaced between 0.1 and 30 cycles per degree (cpd) visual angle. We applied each band-pass filter to the frequency representation of each image, yielding 100 band-pass filtered versions of each image in the frequency domain. We combined the resulting power values of each image in the frequency domain together with the image’s original phase information to compute the corresponding frequency-filtered images using the inverse Fourier transform. This procedure resulted in 100 sets of the stimulus set, band-pass filtered between 0.1 and 30 cpd.

#### Comparing visual representations in infants and adults

We determine whether infants and adults have similar visual category representations using representational similarity analysis (RSA).^89^^,^^90^ The idea is that infants and adults share representations of category if they treat the same categories as similar or dissimilar. We determined this in a two-step process. In a first step, for each age group independently condition-specific multivariate activity patterns (adults: 63 electrodes; infants: 25 electrodes) were compared for dissimilarity. Dissimilarity was determined for all pairwise combinations of conditions, and dissimilarity values were aggregated in so-called representational dissimilarity matrices (RDMs) indexed in rows and columns by the conditions compared (here: 4 × 4 RDMs indexed by the 4 object categories). RDMs thus provide a statistical summary of the similarity and thus representational relations between visual category representations. The RDMs gained from the infant and adult sensor space separately have the same definition and dimensionality and are thus directly comparable. Thus, in a second step, the infant RDM and the adult RDMs are related to each other by determining their similarity.

We applied RSA to two different types of data: evoked responses (i.e., recorded voltage signals) and oscillatory responses (i.e., spectral power). In both cases we re-used the results of the classification analysis described above for the definition of RDMs. Classification accuracy can be interpreted as a dissimilarity measure on the assumption that the more dissimilar activation patterns are for two conditions, the easier they are to classify.^9^^,^^91^ We detail the different RSA procedures below. To reduce visual complexity of the analysis we subsampled the results of the classification analysis by binning them in 10 ms bins.

#### Relating visual category representations in infants and adults based on raw broadband time courses

We investigated whether infants and adults share common visual representations based on broadband responses. As visual representations in adults and infants likely emerge with different time courses, we related their visual representations in a representational similarity time-generalization analysis. As RDMs we used time point specific decoding accuracy matrices (Figure 2A). We first averaged infant RDMs across all participants to increase SNR, resulting in one average infant RDM per time point. We then correlated (Spearman’s *R*) the average infant RDM to each adult (*n* = 20) RDM across all time point combinations. This yielded 20 correlation matrices, indexed in rows and columns by the time points compared (rows: infant time; columns: adult time), indicating when infants and adults share category representations.

#### Relating visual category representations in infants and adults based on frequency-specific power time courses

We investigated whether infants and adults share visual representations in particular frequency bands. As RDMs we used decoding accuracy matrices from the classification analysis based on time-frequency resolved power values. As in this analysis we could neither assume similar time courses, nor similar roles for particular frequencies across infants and adults, we related infant and adult representations in a time-and-frequency-generalization analysis. To do this, we first defined a single aggregate infant RDM by averaging decoding accuracy matrices based on the extent (time and frequency) of the significant cluster in the infant data (Figure 4B) alone. To increase signal-to-noise we only included RDMs whose average across entries in single participants was greater than or equal to 50% decoding accuracy. Note that this criterion is orthogonal to the hypotheses tested and thus does not bias the analysis. We compared (Spearman’s *R*) this single aggregate infant RDM to time- and frequency- resolved RDMs for each participant of the adult sample (*n* = 20), separately for each frequency and time point. This yielded 20 correlation matrices, with rows representing time points and columns representing frequency bins, indicating when and at which frequency infants and adults share category representations.

#### Relating visual representations in infants and adults to computational models

To characterize the format of visual category we related neural representations in infants and results to different computational models using RSA. We constructed model RDMs from computational models (Figure 3A) that represent visual information in different formats. We considered two types of visual computational models: a Gabor wavelet pyramid as a model of low-level feature representations,^47^^,^^92^ and the VGG-19 ^48^ deep convolutional neural network (DNN) trained to categorize object images. Deep neural networks process visual information along a hierarchy of increasing complexity from low to high^93^^,^^94^ that has been shown to match the processing hierarchy of the human brain^46^^,^^95^^,^^96^ and predict human and non-human primate brain activity better than other model class.^12^^,^^97^^,^^98^^,^^99^

To construct model RDMs we first ran all visual stimuli in the study (i.e., 128 object images) through the models and extracted their activation values. More specifically, for the Gabor filter model we extracted a single set of model responses for Gabor wavelets differing in size, position, orientation, spatial frequency and phase. For the DNN we used the MatConvNet toolbox^100^ to extract model neuron activation values from the rectified linear units (Relu) for each layer. We *z*-transformed activation values across stimuli for each stage/layer separately and averaged the transformed values across the 32 stimuli belonging to each of the four categories (i.e., face, body, house and toy), resulting in four category-specific activation values. We formed the patterns into vectors and computed the dissimilarity (1 - Pearson’s *R*) between all pairwise combinations of the four category activation vectors, resulting in a 4 × 4 RDM for each DNN layer of each DNN separately, and one model RDM for the Gabor filter model.

To construct spatial frequency-specific DNN model RDMs, we used an equivalent procedure with the difference that we ran band-pass filtered images through the DNN model separately for each band-pass defined. This resulted in 4 × 4 RDM for each spatial frequency band and DNN layer of the DNN separately.

To construct spatial-frequency specific image-based RDMs, we did not run the images through a model, but the procedure was otherwise equivalent. We directly averaged the pixel values of the filtered images across the 32 stimuli belonging to each of the four categories (i.e., face, body, house, and toy), resulting in four category-specific activation values. We formed the patterns into vectors and computed the dissimilarity (1 - Pearson’s *R*) between all four category activation vectors pairwise combinations, resulting in a 4 × 4 RDM for each frequency band.

To construct neural RDMs that capture category representations well we averaged decoding accuracy matrices from time-resolved category classification (Figures 1C and 1D) in the 95% confidence intervals around peak latency in time-resolved category classification. Our rationale was that peak latency is the time point when categories were linearly best separable and thus their representations most explicit.^101^ To increase signal-to-noise we only included RDMs whose average across entries in single participants was greater than or equal to 50% decoding accuracy. Note that this criterion is orthogonal to the hypotheses tested and thus unbiased. This yielded a single neural RDM for every infant and adult participant. We then related infant and adult neural RDMs to model RDMs using Spearman’s *R*, yielding a single correlation value for each model RDM and participant.

To allow assessing the models’ predictivity with respect to the noise in the data we calculated an upper and lower bound for the noise ceiling,^102^ that is the predictions a perfect model may reach given the noise in the data. This procedure was conducted separately for the infant and the adult sample. To estimate the upper bound we correlated (Spearman’s *R*) each participant’s neural RDM with the mean neural RDM across all participants. To estimate the lower bound we correlated (Spearman’s *R*) each participant’s neural RDM with the mean neural RDM excluding that participant iteratively for all participants. We averaged the results, yielding estimates of the lower and upper noise ceiling for infants and adults.

To reveal whether infants, adults, and the DNN share common representations, we applied variance partitioning using a general linear model (GLM). The procedure was as follows. We first computed two GLMs between the DNN model RDM (i.e., observation) and the infant and adult RDMs (i.e., main regressor), respectively. This revealed the total variance that the model and each age group shared. We then computed two additional GLMs, adding the other age group’s average RDM to the model (i.e., there are two main regressors). From the additional RDMs, we obtained the unique variance of infant and adult RDMs, which was the difference in explained variance after infant and adult RDMs were reduced from the models. From the results of those two types of GLMs, we computed the shared variance that resulted from subtracting the unique variance from the total variance. We applied this analysis for network layers to which infants and adults showed a significant relationship in the correlation analysis, which are layers 3 and 4.

### Quantification and statistical analysis

We used non-parametric statistical inference for random-effects inference to avoid assumptions about the distribution of the data.^103^^,^^104^ We used permutation tests for cluster-size inference, and bootstrap tests for confidence intervals on maxima, cluster onset/offset, and peak-to-peak latency differences. The sample size (*n*) for infants was 40 and for adults 20. Tests were either two- or right-tailed and are indicated for each result separately.

#### Permutation tests

We tested the statistic of interest (i.e., mean decoding accuracy or correlation coefficient in RSA across participants) using sign permutation tests. The null hypothesis was that the statistic of interest was equal to chance (i.e., 50% decoding accuracy, a Spearman's *R* of 0). Under the null hypothesis, we could permute the category labels of the EEG data, which effectively corresponds to a sign permutation test that randomly multiplies participant-specific data with +1 or −1. For each permutation sample, we recomputed the statistic of interest. Repeating this permutation procedure 10,000 times, we obtained an empirical distribution of the data. We converted the original statistic (i.e., correlation coefficient, the decoding time courses, time-time matrices of correlation coefficients or decoding accuracies, and time-frequency decoding matrices) into p values (correlation coefficients), 1-dimensional (time courses), or 2-dimensional (time-generalization or time-frequency) p value matrices.

We controlled the family-wise error rate using cluster-size inference. We first thresholded p value time courses or maps at p < .005 (cluster-definition threshold) to define supra-threshold clusters by contiguity. These supra-threshold clusters were reported significant only if the size exceeded a threshold, estimated as follows: the previously computed permutation samples were also converted to p value time courses/matrices and also thresholded to define resampled versions of supra-threshold clusters. These clusters were used to construct an empirical distribution of maximum cluster size and estimate a threshold of 5% of the right tail of this distribution (i.e., the corrected p values is p < .05).

#### Bootstrap tests

We calculated 95% confidence intervals for the onsets and offsets of significant clusters and the peak latency of the observed effects. To achieve this, we created 1,000 bootstrapped samples by sampling the participants with replacement. For each bootstrap sample, we determined the peak latency as well as onsets of the first significant cluster and the offset of the last significant cluster. This resulted in empirical distributions of peak, onset and offset latencies on which we determined 95% confidence intervals.

To calculate confidence intervals on mean peak-to-peak latency differences, we created 1,000 bootstrapped samples by sampling the participant-specific latencies with replacement. This yielded an empirical distribution of mean peak-to-peak latencies. If the 95% confidence interval did not include 0, we rejected the null hypothesis of no peak-to-peak latency differences. The threshold p < .05 was corrected for multiple comparisons whenever appropriate using FDR correction.

## Data Availability

• Raw and processed data have been deposited at *OSF* and are publicly available as of the date of publication. DOI is listed in the key resources table. • All customized codes have been deposited at *GitHub* and are publicly available as of the date of publication. DOI is listed in the key resources table.

## Supplemental information

Document S1. Figures S1–S4 and Tables S1–S4

Document S2. Article plus supplemental information
